# The effectiveness of Bio-plus2B®, Techno Mos® and their mixture on the rate of egg production, egg characteristics, retention of nutrients and blood metabolites through the early period of production

**DOI:** 10.1186/s12917-024-03900-8

**Published:** 2024-02-14

**Authors:** Fayza M. Salem, A. A. Abd El-Dayem

**Affiliations:** Animal and Poultry Nutrition Department, Desert Research Center, Cairo, Egypt

**Keywords:** Egg production, Probiotic, Prebiotic, Shell thickness

## Abstract

**Background:**

The trend of using probiotic, prebiotic and their mixture as alternative feed additives which works as growth promoters in poultry diets to increase the productive performance and the immunity of the flock still have an importance consideration. So that the aim of this study is to estimate the impact of Bio-plus2B® (probiotic), Techno Mos® (prebiotic) or their mixture (synbiotic) on egg production, egg and shell quality, some blood metabolites and retention of nutrients between 28 and 40 weeks of age. The hens (ISA brown laying) were allocated randomly in 40 cages; 10 cages/treatment with two hens each. The treatments were the control (T1), T2 (Probiotic: 1 g Bio-plus2B® (*Bacillus licheniformis* plus *Bacillus subtilis*)/kg feed), T3 (Prebiotic: 1 g Techno Mos® (Mannanoligosaccarides (MOS) and 1,3 B-glucan) /kg feed) and T4 (Synbiotic: 1 g Bio-plus2B® plus 1 g Techno Mos®/ kg feed).

**Results:**

Hen-day egg production% and mass were significantly increased (*P* < 0.05) with T2 and T4 treatments. The experimental treatments recorded an increase in albumen index, Haugh unit (*P* < 0.01), shell thickness (*P* < 0.05), the retention of crude protein (CP), crude fiber (CF) and ether extract (EE) (*P* < 0.05), plasma globulin, albumin and total protein (*P* < 0.001) versus to the untreated group, while egg weight was not affected. Synbiotic treatment showed a significant (*P* < 0.001) increase in shell calcium content. T3 and T4 treatments were significantly decreased plasma cholesterol (*P* < 0.001) and low density lipoprotein (LDL) (*P* < 0.05). Alanine transaminase (ALT) was significantly (*P* < 0.001) decreased and estradiol hormone was increased (*P* < 0.001) in the experimental groups versus to the control.

**Conclusions:**

It concluded that adding probiotic and/or prebiotic in the early age laying hens diets had beneficial effects for productivity with improving the egg shell thickness.

## Background

Some feed additives such as probiotics and prebiotics had been introduced as good sources to enhance the productivity of layers hens. Probiotic is a substance that contains microorganisms or bacteria that have a positive influence on improving the gut microbial balance [1]. Prebiotic is non-digestible food ingredient that affects the host by selectively stimulating the growth and/or activity of beneficial bacteria in the intestinal tract [2]. Synbiotic is the combination of probiotics and prebiotics that considered as antimicrobial growth promoters and have positive effects on the metabolic processes [3], and thus improve feed conversion ratio and egg production [4]. The supplementation of probiotic in laying hens’ diet significantly increased estradiol hormone level during the laying early age of layers hens [5] which affecting the growth, development, maturation and functioning of reproductive tracts [6]. Addition of probiotic and/or prebiotic provided an increment in the egg production, weight and mass, shell weight, shell thickness, albumen quality and reduced yolk cholesterol [7, 8]. Using of probiotics, prebiotic and their combination recorded significantly increase in egg production, mass and weight and feed efficiency [9]. The aim of this work is to study the effectiveness of probiotic, prebiotic or synbiotic as feed additives on the egg production, egg quality characteristics during the early stage of production. Then, the hypothesis to be tested is that probiotic, prebiotic or synbiotic increases the egg production during its early stage.

## Methods

### The bird’s management and the experimental treatments

This experiment was achieved in south Sinai experimental research station (Ras-Suder City) which belongs to the Desert Research Center. Eighty hens of ISA Brown breed (28–40 weeks old) were allocated randomly in 40 cages; 10 cages/ treatment and with 2 hens each. The hens were housed in wire cages of triple deck batteries. The experimental treatments were the control (T1), T2 (Probiotic: 1 g Bio-plus2B®/kg feed), T3 (Prebiotic: 1 g Techno Mos® /kg feed), T4 (Synbiotic: 1 g Bio-plus2B® plus 1 g Techno Mos®/ kg feed). The continuous light duration was 16 h. Table 1 represented the diet formula [10]. It was iso-nitrogenous (18% CP) and iso-caloric (2670 Kcal ME/Kg). Feed and clear drinking water were continuously available. The indoor temperature (21.9ºC ± 0.23) and relative humidity (RH% 50.5 ± 0.89) were recorded as average. The chosen feed additives are Bio plus 2B® (*Bacillus licheniformis* CH 200/DSM 5749 1.6*10^9^ CFU/g and *Bacillus subtilis* CH 201/DSM 5750 1.6*10^9^) and Techno Mos® (Mannanoligosaccarides (MOS) and 1,3 B-glucan that is derived from the cell wall of the yeast *Saccharomyces cerevisiae*).

**Table 1:** Composition and calculated values of the experimental diet

Ingredients	%
Yellow corn	58.40
Soybean meal 44%	20.50
Corn gluten meal 60%	5.00
Limestone	7.50
Di-Calcium phosphate	1.70
Wheat bran	6.00
DL-Methionine	0.30
Salt	0.30
Vit&Min. Premix*	0.30
Total	100
Calculated Values	
Crude protein%	18.03
ME (Kcal/kg)	2677.6
Calcium%	3.30
Available P%	0.43
DL-Methionine%	0.62
L-Lysine%	0.79

*Vitamins and minerals premix, each 2.5 kg contain: Vit A 10,000,000 IU, Vit D3 2,000,000 IU, Vit E 10 g, Vit K 1000 mg, Vit B12 10 mg, Vit B1 1000 mg, Vit B2 5000 mg, Vit B6 1.5 g, Pantothenic acid 10 g, Niacin 30 g, Folic acid 1 g, Biotin 50 mg, Iron 30 g, Manganese 70 g, Choline chloride 10 g, Copper 4 g, Zinc 50 g, Selenium 100 mg and Iodine 300 mg

### The experimental measurements

The consumption of feed was recorded weekly. The calculation of egg mass (g/hen/day) was by using egg weight and egg number. FCR (g feed intake/g egg mass) considered as the amount of feed consumed divided by egg mass. At the end of the experiment, ten eggs were taken from each treatment for measuring egg and shell quality traits (egg weight, albumen% and index, yolk%, Haugh unit, shell%, shell thickness and shell Ca%). Albumen, yolk and shell% was calculated as their weights relative to egg weight. Shell Ca% was estimated by Inductively Coupled Argon Plasma, ICAP 6500 Duo, Thermo Scientific, England- 1000 mg/L multi-element certified standard solution, Merck, Germany was considered as stock solution for instrument standardization.

Haugh unit was calculated [11]:

Haugh unit = 100 x log (H + 7.57–1.7 x W^0.37^).

Where: H = Albumen height, W = Egg weight.

Albumen index= (Albumen height/Albumen diameter)*100.

Shell thickness (ST) was measured without membrane using micrometer.

### Digestibility trail

Fresh samples of feces were taken every 24 h from 5 hens for each treatment during the last three consecutive days of the study. Feed consumption and feces were weighed and dried at 65ºC till constant weight and conserved for the approximate analysis of feed and feces for dry matter (DM), organic matter (OM), crude protein (CP), crude fiber (CF) and ether extract (EE).

### Blood biochemical profiles

At the last day of this study, five hens were taken at random from each experimental treatment to take the blood samples from brachial wing vein of the alive hens (without anaesthetize or euthanize) and they were immediately centrifuged at 3000 rpm for 20 min, and then plasma was stored at -20ºC for later analysis. The hens were released after taking the blood samples. Blood parameters included total cholesterol, HDL, LDL, triglycerides, total protein, albumin, alanine transaminase (ALT), aspartic transaminase AST, and estradiol hormone (E2). All parameters were determined colorimetrically by using BioMed diagnostic kits except estradiol hormone (E2) which determined by immunoassay analyzer with using iFlash kits. Globulin resulted from subtracting albumin from total protein.

### Data analysis

The data was analyzed by using simple one-way analysis of variance by SAS program [12] according to this model: Yij = µ + T_i_+ e_ij_,

Where: µ = General average, T_i_ = Random effect of treatment (i = 1, 2, 3 and 4) and e_ij_ = Error. The separation among means was occurred by using Duncan test [13].

## Results

### Productive performance

The data of hen-day egg production%, egg weight, and egg mass, feed consumption and FCR during the experimental period are shown in Table 2. Egg production% and egg mass in probiotic (T2) and synbiotic (T4) groups were significantly (*P* < 0.05) greater than in prebiotic (T3) and control (T1) groups. The differences between control and prebiotic treatment were non-significant. Regarding egg weight, feed consumption and FCR, there were non-significant differences among treatments.

**Table 2:** Impact of Bio-plus2B®, Techno Mos® and their mixture on the productivity of laying hens

			Treatments			
Item	T1	T2	T3	T4	SE	*P* value
Hen day egg production%	66.91^b^	80.95^a^	65.71^b^	83.10^a^	3.63	0.016
Egg weight (g)	63.26	64.15	65.09	64.81	0.654	0.274
Egg mass (g)	42.34^b^	51.99^a^	42.83^b^	54.53^a^	2.64	0.022
Feed consumption (g/hen/day)	103.23	104.57	99.01	101.78	12.94	0.991
FCR (FI/Emass)	2.42	1.99	2.29	1.87	0.19	0.222

a,b,c Means within the same row showing different letters are significantly different (Duncan test), SE = Stander error, FCR = feed conversion ratio

### Egg and shell quality traits

Table 3 represents the effectiveness of the experimental treatments on egg and shell quality traits (egg weight, albumen% and index, yolk%, haugh unit, shell%, shell Ca% and shell thickness (mm)). Albumen index, haugh unit (*P* < 0.01) and shell thickness (*P* < 0.05) were significantly increased by the experimental treatments versus to the untreated group, while egg weight, albumen%, yolk% and shell% was not affected. Shell Ca% was significantly increased (*P* < 0.001) by synbiotic treatment (T4) versus to the other groups.

**Table 3:** Impact of Bio-plus2B®, Techno Mos® and their mixture.on egg and shell quality traits

			Treatments			
Item	T1	T2	T3	T4	SE	*P* value
Egg weight (g)	63.04	66.70	65.63	64.37	1.17	0.164
Albumen%	63.20	65.94	63.49	62.92	1.73	0.616
Yolk%	24.24	24.74	23.87	24.24	0.674	0.841
Albumen index	9.90^b^	11.20^ab^	11.97^a^	12.37^a^	0.479	0.004
Haugh unit	79.94^b^	85.23^a^	88.45^a^	89.92^a^	1.75	0.002
Shell%	11.95	12.38	12.17	12.88	0.441	0.501
Shell Ca%	30.68^b^	30.53^b^	30.57^b^	37.42^a^	0.843	0.0001
ST (mm)	0.444^b^	0.494^ab^	0.526^ab^	0.571^a^	0.031	0.040

a,b,c Means within the same row showing different letters are significantly different (Duncan test), SE = Stander error

ST = shell thickness

### Retention of nutrients

The impact of probiotic (T2), prebiotic (T3) and synbiotic (T4) on the retention of dry matter (DM), organic matter (OM), crude protein (CP), crude fiber (CF) and ether extract (EE) are shown in Table 4. A significant increase (*P* < 0.05) in the retention of CP and CF was observed with the experimental treatments versus to the untreated group, while EE retention was tended to increase. Regarding DM and OM retention was not affected by the experimental groups.

**Table 4:** Impact of Bio-plus2B®, Techno Mos® and their mixture.on retention of nutrients%

			Treatments					
Item	T1	T2	T3			T4	SE	*P* value
DM Retention%	75.06	78.77	78.73			75.76	3.13	0.76
OM Retention%	76.27	79.82		79.84	77.87		2.92	0.79
CP Retention%	64.05^b^	77.72^a^		79.16^a^		74.88^a^	3.13	0.03
CF Retention%	69.69^b^	87.11^a^		71.20^b^		81.18^ab^	3.63	0.02
EE Retention%	67.02^b^	76.45^ab^		81.33^a^		70.53^ab^	3.75	0.08

a,b,c: Means within the same row showing different letters are significantly different (Duncan test), SE = Stander error

### Blood biochemical profiles

Table 5 represented the effectiveness of using probiotic, prebiotic and their mixture to hens’ diets on blood metabolites. High significant increase in plasma total protein, globulin (*P* < 0.001) and albumin (*P* < 0.01) with probiotic and synbiotic treatments was observed, while the increase in these parameters regarding prebiotic versus to the untreated group was non-significant. T3 and T4 treatments significantly decreased total cholesterol (*P* < 0.001) and LDL (*P* < 0.05), while the numerical increase in HDL was observed with probiotic and synbiotic treatments. Regarding triglycerides and AST, the effect of the experimental treatments was non-significant. Likewise, ALT was significantly (*P* < 0.001) decreased and estradiol hormone (E2) was increased (*P* < 0.001) in the experimental groups especially with synbiotic group versus to the untreated group.

**Table 5:** Impact of Bio-plus2B®, Techno Mos® and their mixture.on some plasma parameters

			Treatments			
Item	T1	T2	T3	T4	SE	*P* value
Total protein (g/dl)	5.50^b^	14.67^a^	7.10^b^	13.00^a^	0.92	0.0003
Albumin (g/dl)	1.20^b^	1.83^a^	1.37^b^	1.83^a^	0.122	0.0124
Globulin (g/dl)	4.30^b^	12.83^a^	5.73^b^	11.17^a^	0.946	0.0005
Cholesterol (mg/dl)	158.60^a^	165.50^a^	92.60^b^	151.37^a^	6.05	0.0002
HDL (mg/dl)	16.00	17.00	10.67	18.67	3.202	0.377
LDL (mg/dl)	21.37^a^	23.27^a^	16.47^ab^	10.27^b^	2.803	0.044
Triglycerides (mg/dl)	432.60	476.90	439.80	657.20	87.70	0.299
ALT (u/l)	20.00^a^	17.00^b^	16.67^b^	14.00^c^	0.441	0.0001
AST (u/l)	235.00	309.50	266.33	220.67	57.78	0.716
Estradiol hormone (pg/ml)	113.94^c^	166.97^c^	252.67^b^	899.00^a^	21.61	0.0001

a,b,c: Means within the same row showing different letters are significantly different (Duncan test), SE = Stander error

## Discussion

The results indicated positive and significant (*P* < 0.001) superiority of probiotic and synbiotic treatments on hen-day egg production and mass, over prebiotic and control groups. The improvement in egg production in this experiment may be back to the antimicrobial growth promoters’ effect of probiotics and prebiotics, which have positive effects on the metabolic processes and thus the nutrient utilization [3]. Also, the synergism between probiotic and prebiotic together actually lead to better nutrients utilization, metabolism and good absorption. These results were in conformation with those who concluded that egg laying rate and egg mass in probiotic and synbiotic groups were significantly higher than the untreated group [7–9]. Adding *Bacillus licheniformis* and *Bacillus subtilis* [14] and the combination of *Lactic acid bacteria, Bacillus subtilis* and *Saccharomyces* [5] in diets of layers hens significantly increased the egg production rate. The numerical improvement in FCR with synbiotic and probiotic treatments may be due to the useful impacts of probiotic and/or prebiotic in increasing the nutrient absorptive surface area in the intestine [15]. Similar results reported non-significant improvement in FCR regarding T2 and T4 treatments and insignificant differences in feed intake [16]. The produced eggs with high value of albumen index and haugh unit are considered as a good egg quality indicator. Haugh unit is a measure of protein quality and freshness of eggs [17]. The United States Department of Agriculture (USDA) classified the eggs in descending order based on its desirability as AA (72 or more) which agrees with our results, A (71 − 60), and B (59 − 31) according to Haugh unit [11]. The excellent score is 90 or above, 70 is good and below 60 is rejected. Probiotic supplementation increased albumen height and Haugh unit compared with the control [5]. The increase in shell thickness and shell Ca% probably related to the existence of prebiotics which fermented by the intestinal microflora or probiotic bacteria and increased the produced short chain fatty acids and reduced luminal pH [18]. Low pH increased calcium solubility and thus absorption [19]. This indicated that synbiotic addition provided favorable acidic environment inside the intestine, which helped in improving calcium digestion and absorption. Calcium retention was improved when laying hens diet contained *Lactobacillus* [20]. The maximum increase in shell thickness was observed with *Bacillus subtilis* [8, 21] and probiotic [5] versus to the untreated group. *Bacillus subtilis* represented a non-significant effect on yolk% and albumen% versus to the untreated group [21]. In our study, the improvement in digestibility of EE in all the experimental treatments versus to the untreated group probably back to the high content of CF retention% which increases the HCl and bile salts production where the production of HCl and bile salts that emulsify fats is enhanced when low-fiber diets are supplemented with adequate amounts of fiber [22] such as prebiotic in our study. Likewise, probiotic bacteria can digest fiber and affect the metabolic processes of the beneficial bacterial colonies inside the layer’s intestine which lead to the improvement in the digestion coefficient of nutrients. The CP and EE showed significantly higher digestibility of the diet supplemented with *Saccaromyces cerevisiae* fed to broilers [23] and *Entercoccus faecium* addition in layers hens diets improved the nutrients digestibility [24]. Adding mannanoligosaccharides (MOS) in layers hens’ diet significantly increased the digestibility coefficient of DM, CP and EE [25]. The low blood cholesterol effect of prebiotics may be related to the production of short chain fatty acids (SCFA) from prebiotic fermentation by intestinal microflora, which contained acetic, propionic and butyric acids [26]. SCFA could reduce the synthesis of hepatic cholesterol [27] and stimulate bile acid synthesis [28], which could decrease the level of blood cholesterol. Prebiotic and/or synbiotic addition in laying hens’ diet at 36 weeks of age significantly reduced total cholesterol and LDL, while HDL and triglycerides levels were not affected [29]. Synbiotic treatment significantly increased total proteins, albumin and globulin [30]. The quantity of ALT and AST in the serum considered as an indicator of organ or tissue damage degree. The evaluation of avian hepatic function can be occurred by using the concentration of ALT and AST due to their synthesis in the liver [31]. Laying hens fed on prebiotic, probiotic or their mixture diet recorded low concentration of ALT than the control [29]. Estradiol (E2) hormone is a main reproductive hormone affecting growth, development, maturation and functioning of reproductive tracts [6]. Also, the synthesis of albumen proteins in the oviduct were occurs by estradiol hormone [32] and affects liver function and stimulates egg yolk precursors [33]. Probiotic addition in laying hens’ diet significantly increased the level of estradiol hormone versus to the untreated group [5, 34].

## Conclusion

Adding probiotic (Bio-plus2B®), prebiotic (Techno Mos®) or synbiotic in laying hens rations significantly increased the rate of egg production, egg mass, egg shell thickness and some blood parameters such as estradiol hormone through the early period of production.

## Data Availability

All data generated and analyzed during this study are included in this published article.
